# Impact of Multidecadal Climate Variability on United Kingdom Rickets Rates

**DOI:** 10.1038/s41598-017-16058-1

**Published:** 2017-11-17

**Authors:** Haris Majeed, G. W. K. Moore

**Affiliations:** 1 Institute of Medical Sciences, University of Toronto, 1 King’s College Circle, Toronto, ON M5S 1A8 Canada; 2 Department of Diagnostic Imaging, The Hospital for Sick Children, 555 University Ave, Toronto, ON M5G 1X8 Canada; 3 Department of Physics, University of Toronto, 60 St. George Street, Toronto, ON M5S 1A7 Canada; 4 Department of Chemical & Physical Sciences, University of Toronto Mississauga, 3359 Mississauga Road, Mississauga, ON L5L 1C6 Canada

## Abstract

Children who receive inadequate exposure to sunlight have reduced levels of vitamin D, resulting in rickets, a disease that is characterized by bone deformity, stunted growth, and long term pronounced disability. The United Kingdom rickets incidence rates declined from the early 1960’s to mid-1990’s, after which there was a dramatic increase. The reason for this change is not well understood. Here we show that an important low frequency mode of climate variability, the Atlantic Multidecadal Oscillation (AMO), with a period of ~60–80 years, has an impact on rickets incidence rates in the United Kingdom through changes in sea level pressure, cloud cover and sunshine duration. This research highlights the important role that multidecadal climate variability can play in human morbidity and suggests that future changes in the AMO may modulate rickets incidence rates throughout the United Kingdom.

## Introduction

The primary source of vitamin D is from the sun’s exposure to the human skin, which is essential to both adults and children for the metabolism of calcium and phosphorous^1^. In the absence of vitamin D, only 10–15% of dietary calcium and ~60% of phosphorus is absorbed in the body^2^. This deficiency of vitamin D in children was originally referred to as “the English disease”, which is now internationally recognized as rickets^3^. Rickets results in the absence of phosphate at the growth plate and mineralising bone surfaces in children^3^. For children with rickets, the long term effects of this disease can cause a reduction of bone size and mass predisposing to osteoporotic fracture^3^. Vitamin D deficiency has now approached near-pandemic proportions, with researchers estimating that approximately one billion individuals worldwide lack the sufficient amount of vitamin D, thus this has been a rising concern^2–4^.

The annual hospitalization rickets incidence rates for children younger than 15 years in the United Kingdom are now the highest in past five decades^5^. In 1994, the United Kingdom government issued guidelines for vitamin D supplementation to clinicians; however, a survey conducted in 2001 acknowledged the continued presence of vitamin D deficiency in children^3,6^. Numerous reasons for vitamin D deficiency in children have been documented. Recent surveys have found a strong relationship between the vitamin D status of newborn infants and that of their mothers, hence suggesting that maternal deficiency leads to overt bone disease starting before birth^7,8^. There exist limited children’s food options which provide a rich intake of vitamin D^4^. To overcome this, additional supplements are needed to maintain adequate vitamin D levels in the body^4^. However, the primary cause of rickets is the absence or diminished levels of sun exposure, which heavily depends on the patient’s location of residence^3,5,9^. In the Northern hemisphere, the amount of exposure to sunlight is reduced for children living at latitudes greater than 35° (ref.^3^). Children in high latitude countries, such as the United Kingdom, receive low levels of vitamin D during the winter^9,10^; while during the summer, they receive sufficient but suboptimal vitamin D levels with ~0.22 hours/day (~13 minutes/day) of sun exposure^11^.

The Atlantic Multidecadal Oscillation (AMO) is a climate oscillation with a period of ~60–80 years that is related to coherent low frequency variability in sea surface temperatures across the North Atlantic^12–15^. The AMO modulates a number of important climate processes such as Mississippi stream flow, Sahel rainfall intensity and North Atlantic hurricane formation^12,13^. In addition, the positive (warm) AMO phase is related to a decrease in average sea level pressures with increased rainfall over Northwest Europe, and vice-versa in the negative (cool) phase^14,15^. Given the relationship between sea level pressures and cloudiness^16–19^, it is therefore likely that the AMO may also modulate sunshine duration in the United Kingdom. In this paper, we investigate the connection between this low frequency climate mode, AMO and rickets incidence rates. Previous research has failed to recognize the important connection between low frequency climate variability and an important debilitating disease.

## Results

**Figure 1:**
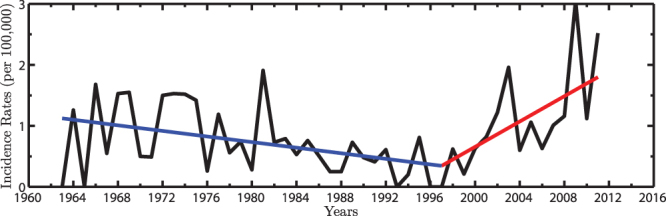
Rickets Incidence Rates. Oxford rickets incidence rates (black), with a decline in rickets from 1963–1997 (−0.23 incidences/100,000/decade, >99% CL), followed by a sharp increase in rickets from 1997–2011 (1.04 incidences/100,000/decade, >99% CL). A breakpoint in the year 1997 was statistically determined by a minimum found in the root mean square error.

The most well documented rickets incidence rates for the United Kingdom are from Oxford National Health Service (NHS) region (population ~2.5 million). The data extends back to the early 1960’s^5^ (Fig. 1). Please refer to ‘Methods’ for more information on this dataset. Rickets incidence rates in the United Kingdom were found to be in decline prior to the mid-1990’s, with a sudden increase occurring after that^5^. A piecewise linear fit was applied to the data to determine the statistical significance of the trend before and after this transition. The breakpoint year was determined based on a minimum in the root mean square error, which in this case was found to be 1997 ± 3 years. In addition, using a Monte Carlo significance test which takes into account a reduction in the degrees of freedom arising from temporal autocorrelation in the rickets incidence rates (See Methods). It was found that between 1963–1997, the median in rickets incidence rates were 0.56 incidences/100,000, with a statistically significant trend of −0.23 incidences/100,000/decade (>99% CL). Over the period from 1997–2011, rickets incidence rates increased to a median of 1.01 incidences/100,000, with a statistically significant trend of 1.04 incidences/100,000/decade (>99% CL).

**Figure 2:**
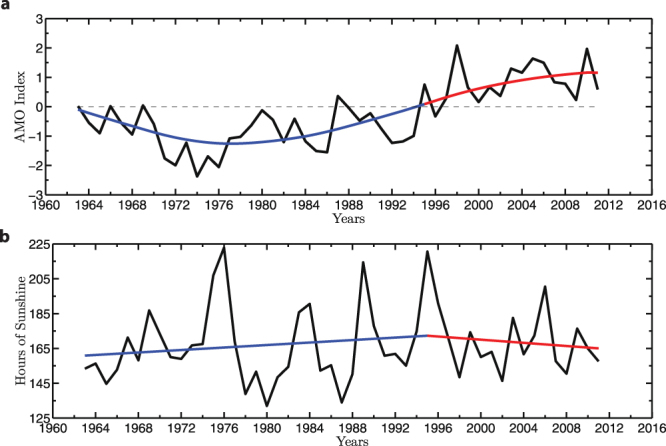
AMO Index and Summer Sunshine. (**a**) The annual AMO index (black) with its low frequency variability (blue: 1963–1995, red: 1995–2011). (**b**) Total summer (June-August) mean duration of United Kingdom sunshine (black), with an increasing number of hours from 1963–1995 (2.36 hours/month/decade, >99% CL), followed by a decline of sunshine from 1995–2011 (−4.02 hours/month/decade, >99% CL). A breakpoint in the year 1995 was statistically determined by a minimum found in the root mean square error.

The annual AMO index along with its low frequency variability (Fig. 2a) shows a sign change (i.e. negative to positive phase) that occured in the mid-1990’s. Prior to mid-1990’s, the average summer sunshine duration in the United Kingdom had a statistically significant increase of 2.36 hours/month/decade (>99% CL), with a statistically significant decline in sunshine of −4.02 hours/month/decade (>99% CL) occurring after this period (Fig. 2b). The change of phase seen in the AMO index is consistent with the changing trend of sunshine duration in the United Kingdom.

**Figure 3:**
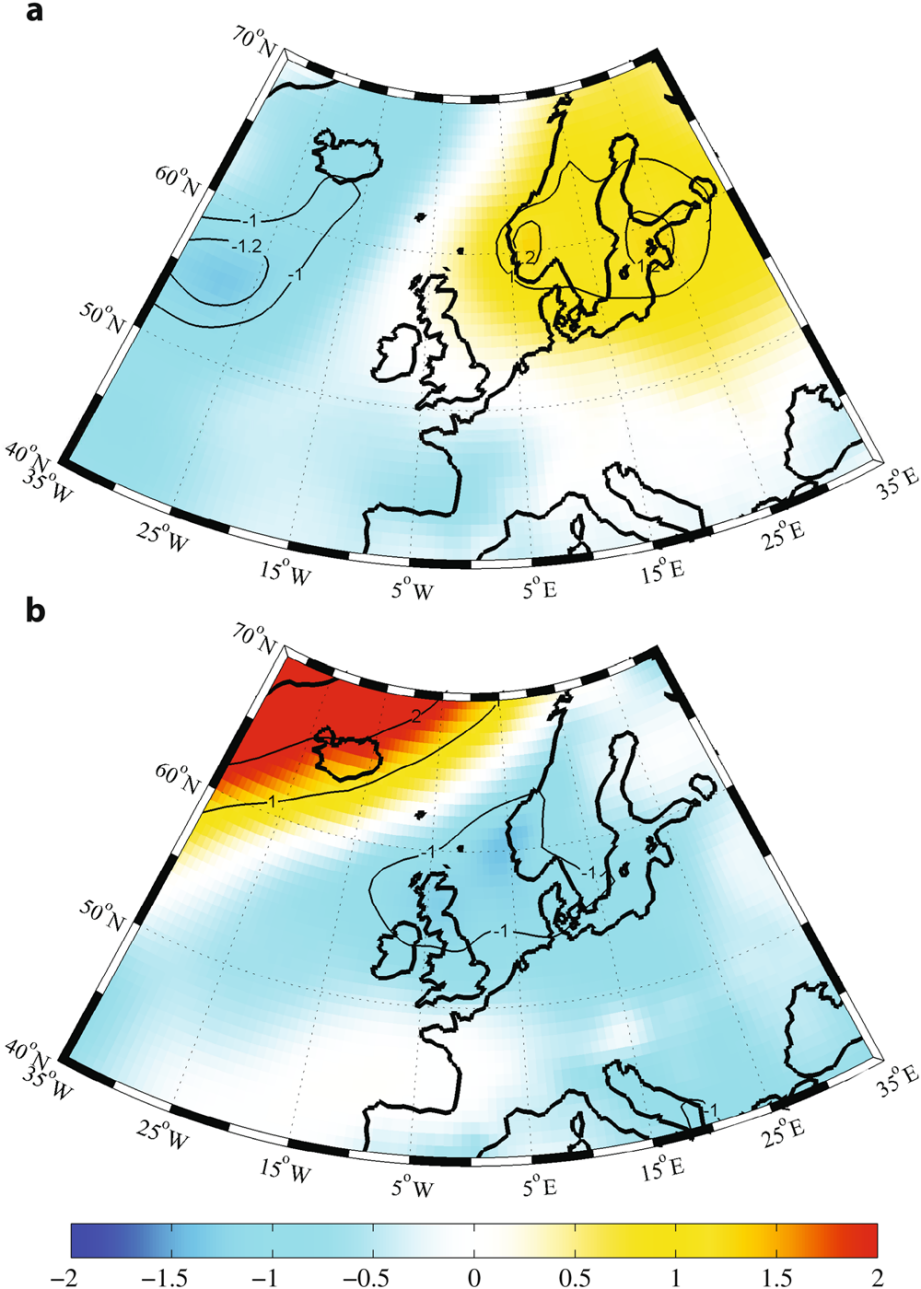
Spatial Summer Sea Level Pressure Trends. (**a**) Spatial summer sea level pressure trends (millibars/decade) from 1979–1997. (**b)** Spatial summer sea level pressure trends (millibars/decade) from 1997–2011. The significant trends (>95% CL) were calculated using Monte Carlo simulations, which have been contoured. The ERA-Interim dataset was used along with MATLAB R2012b (https://www.mathworks.com/products/matlab.html).

As shown in Fig. 2, the change in AMO phase is associated with changes in sea level pressure as well as the duration of United Kingdom summer sunshine. Hence additional evidence in the changing nature of rickets incidence rates is provided by sea level pressure trends before and after the mid-1990’s (Fig. 3). Spatial summer sea level pressure trends before 1997 (Fig. 3a) indicate a statistically significant increase of 1.0 millibars/decade with a center over to the East of the British Isles. Since the late 1970’s to mid-1990’s, summer sea level pressures over the United Kingdom have caused drier conditions, subsequently resulting in a reduction of cloud cover and precipitation^16,17^. On the other hand, average summer spatial sea level pressure trends from 1997–2011 (Fig. 3b) are found to be negative and statistically significant primarily over the North Sea and Northeastern British Isles. This negative or low sea level pressure trend creates strong cyclonic wind flow around the British Isles, bringing increased cloud cover and blocking summer sunshine over England during this period.

**Figure 4:**
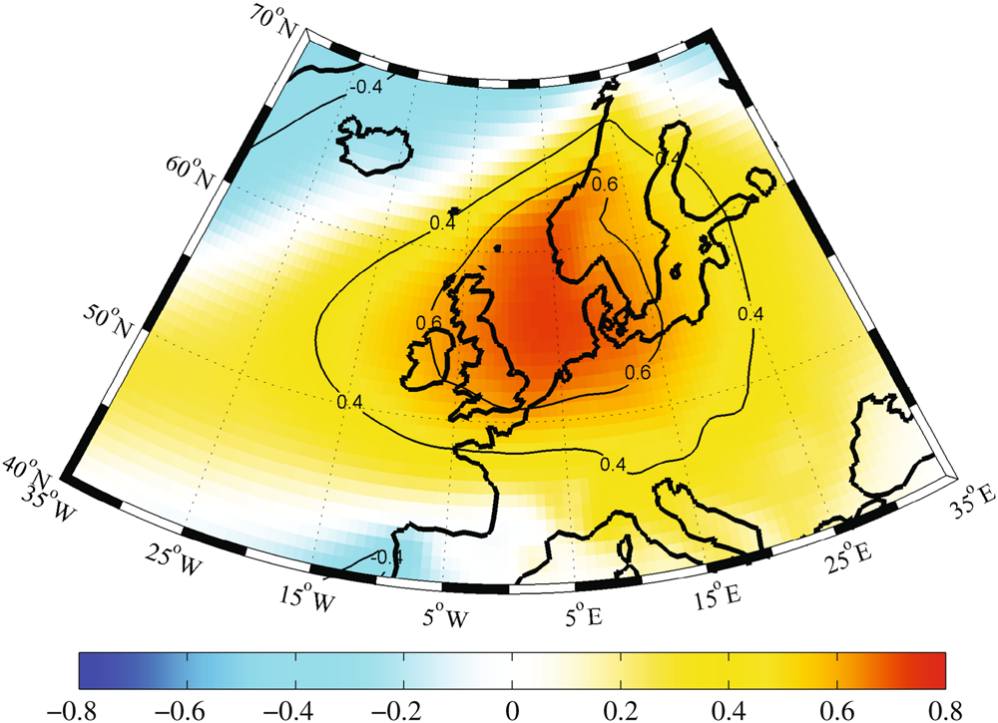
Spatial Summer Correlation. United Kingdom mean summer sunshine correlated to spatial summer sea level pressures from 1979–2011. The significant correlations (>95% CL) were calculated using Monte Carlo simulations, which have been contoured. The ERA-Interim dataset was used along with MATLAB R2012b (https://www.mathworks.com/products/matlab.html).

Furthermore, the correlation between United Kingdom summer sunshine to sea level pressures from 1979–2011 (Fig. 4) illustrates this statistically significant relationship over the British Isles and the North Sea. From Fig. 4 it is clear that an increase or decrease in sunshine duration is positively associated to an increase or decrease in sea level pressures, respectively. This association explains ~36% of the variance in the variability of summer sunshine due to sea level pressures over the United Kingdom.

## Discussion

In general, meteorological observations indicate that low sea level pressures increase cloud cover and precipitation while decreasing temperatures with the opposite occurring for high sea level pressures^18,19^. The observed increase in cloud cover after mid-1990’s is consistent with our findings of a decline in United Kingdom average summer sunshine duration. In the past few decades Western Europe has exhibited increased cloud cover during the summer seasons, causing cooler temperatures^20^. This shift is thought to be associated with the AMO^12–15^. In addition, a recent eastward shift of the location in the Icelandic Low, a major atmospheric circulation system impacting the climate of Western Europe^21^, has resulted in increased summer precipitation and lower temperatures in the United Kingdom^15^.

We propose the decline in rickets incidence rates, followed by an increase after the mid-1990’s can be explained by a low-frequency variability associated with the AMO. From early 1960’s to mid-1990’s, the AMO remained in a negative phase which resulted in high summer sea level pressures, reducing cloud cover and precipitation over the British Isles, United Kingdom, as well as England^14,15^. As a result, an increasing duration of sunlight was able to reach the surface, leading to a reduction in rickets incidence rates. After 1997 the rise in rickets incidence rates can be explained through the AMO change in phase from negative to positive. This positive phase of the AMO, which is present till today, resulted in a tendency for increased lower sea level pressures and decreased sunshine duration over the United Kingdom^14,15^. The climate phenomena related to the switching of AMO phase from negative to positive is consistent with our results for spatial summer sea level pressure trends.

As mentioned above, in the United Kingdom ~0.22 hours/day or ~6.5 hours/month of sun exposure is needed for healthy vitamin D production^11^. This suggests that the recent decline in sunshine duration is clinically important with respect to vitamin D production. Additionally, cloud cover has the potential of blocking up to 99% of vitamin D metabolism in humans^22^.

Although our research suggests that the low frequency variability of the AMO plays a role in United Kingdom rickets incidence rates, other factors such as changes in hospital admission thresholds, diagnostic practices, children’s diet, and inherited disorders can also contribute to rickets incidence rates^3,5^. The AMO has now been in a positive phase for ~20 years. Assuming that the AMO has a period of ~60–80 years, this positive phase will persist for another 10–20 years from the current decade. This suggests that a further increase in the United Kingdom rickets incidence rates will occur over the next one to two decades.

In 1994, despite the United Kingdom’s government guidelines for vitamin D, the England’s chief medical officer in January 2011 has advised children aged six months to five years towards consuming vitamin D supplements^23^. Regardless of children’s increase in vitamin D supplementation since 2011, the rise in rickets incidence rates still remains unclear^3,5^. While the ongoing efforts of clinical and government interventions have been established^24^, environmental and climate related phenomena often seem to be ignored.

## Methods

### Rickets Incidence Rates

The rickets data were obtained from Prof. Dr. Michael Goldacre director of the Health-Care Epidemiology Unit at the University of Oxford. This data was first published in *The Lancet* ^5^ in 2014 and it was the first to depict a historical perspective of United Kingdom rickets incidence rates over nearly the past five decades. The rickets time series from 1963–2011 was constructed by analyzing annual hospital admissions for children with rickets (ages <15 years) in the Oxford National Health Service (NHS) region of England. This region includes the following counties; Berkshire, Buckinghamshire, Oxfordshire, Northamptonshire, with a total mean population of ~2.5 million^5^. The time series is based on patient records according to the International Classification of Diseases (ICD) codes. Hence the time series was based on the ICD codes for ‘active rickets’; ICD7 code 283, ICD8 code 265.0, ICD9 code 268.0, and ICD10 E55.0. ICD7 covers the period of 1955–1964, ICD8 from 1965–1974, ICD9 from 1975–1989, and ICD10 from 1990 till today. The rickets incidence rates depicted a change from low to high rates during the mid-1990’s^5^. Hence, a piecewise linear fit was applied. The year of the breakpoint was determined by computing the root mean squared error between the years 1990–2000. During this time period, the minimum root mean squared error was 0.527 found in the year 1997. Consequently, 1997 was chosen as the breakpoint for the piecewise linear fit ( ± 3 year uncertainty).

### AMO Index and United Kingdom Sunshine

The annual AMO index was obtained from the National Oceanic and Atmospheric Administration (NOAA), which is the annual mean sea surface temperature field over the domain from 0°N-70°N and 75°W-7°W, the same domain used in the original definition of the AMO index^12^. The United Kingdom sunshine data was obtained from Met Office, averaged for summer months (June-August). Given United Kingdom’s location in the mid-latitudes, the duration of sunlight is less during the winter, ~50 hours/month, than during the summer, ~170 hours/month. As a result, we chose to focus on the summer season where both sunlight duration and vitamin D levels are higher^25^. The breakpoint year in the sunshine data was determined to be 1995 (±2 year uncertainty) using the same method for rickets incidence rates.

### Time Series Analysis

The Oxford rickets incidence rates as well as sea level pressures have a temporal autocorrelation from one year to the next, resulting in red noise spectra that are characterized by a reduction in the number of degrees of freedom^26^. For this reason the statistical significance (>95% CL) of the piecewise linear fits, spatial trends and correlations were determined from a Monte Carlo simulation, generating 10,000 synthetic time series with the same spectral characteristics as the original time series^13^. Furthermore, the Singular Spectral Analysis (SSA) was used to reconstruct the low frequency variability in the annual AMO index, since this technique employs data adaptive basis functions to separate a time series into statistically independent components that maximize the variability that each basis function describes^27^.

### Spatial Grid Point Trends and Correlations

For spatial grid point trends and correlations, the summer (June-August) sea level pressure ERA-Interim (ERAI) reanalysis dataset was used^28^. The ERAI is the latest global atmospheric reanalysis produced by the European Centre for Medium Range Weather Forecasts (ECMWF), with a resolution grid of 0.75° by 0.75°, which extends through a period of 1979 till today^28^. The spatial trends and correlations were restricted to the longitude domain of 35°W-35°E and a latitude domain of 40°N-70°N. All figures were produced using MATLAB R2012b.
